# Factors associated with patient-reported experiences and outcomes of substance use disorder treatment in Cape Town, South Africa

**DOI:** 10.1186/s13722-022-00289-3

**Published:** 2022-02-02

**Authors:** Bronwyn Myers, J. Randy Koch, Kim Johnson, Nadine Harker

**Affiliations:** 1 Curtin enAble Institute, Faculty of Health Sciences, Curtin University, Kent Street, Bentley, WA 6102 Australia; 2 Alcohol, Tobacco and Other Drug Research Unit, South African Medical Research Council, Cape Town, South Africa; 3 Division of Addiction Psychiatry, Department of Psychiatry and Mental Health, University of Cape Town, Cape Town, South Africa; 4 Department of Psychology, Virginia Commonwealth University, Richmond, VA USA; 5 School of Public Health, University of Cape Town, Cape Town, South Africa

**Keywords:** Treatment quality, Access, Substance use outcomes, Patient-reported outcome measure (PROM), Patient-reported experience measures (PREM), Low-and middle-income country, Quality improvement

## Abstract

**Background:**

Interventions are needed to improve the quality of South Africa’s substance use disorder (SUD) treatment system. This study aimed to identify factors associated with patient-reported suboptimal access, quality, and outcomes of SUD treatment to guide the design of targeted quality improvement initiatives.

**Method:**

We analysed clinical record and patient survey data routinely collected by SUD services in the Western Cape Province, South Africa. The sample included 1097 treatment episodes, representing 32% of all episodes in 2019. Using multivariate logistic regression, we modelled socio-demographic, substance use and treatment correlates of patient-reported suboptimal access to, quality and outcomes of SUD treatment.

**Results:**

Overall, 37.9% of patients reported substantial difficulties in accessing treatment, 28.8% reported suboptimal quality treatment, and 31.1% reported suboptimal SUD outcomes. The odds of reporting poor access were elevated for patients identifying as Black/African, in residential treatment, with comorbid mental health problems, and longer histories of substance use. Length of substance use, comorbid mental health problems, and prior SUD treatment were associated with greater likelihood of reporting suboptimal quality treatment. Patients with comorbid mental health problems, polysubstance use, who did not complete treatment, and who perceived treatment to be of poor quality were more likely to report suboptimal outcomes.

**Conclusion:**

This study is among the first to use patient-reported experiences and outcome measures to identify targets for SUD treatment improvement. Findings suggest substantial room to improve South African SUD treatment services, with targeted efforts needed to reduce disparities in outcomes for patients of Black/African descent, for those with comorbid mental health problems, and for patients who have chronic substance use difficulties. Interventions to enhance the relevance, appropriateness, and acceptability of SUD services for these patient sub-groups are needed to improve system performance.

## Background

While untreated substance use disorders (SUD) contribute substantially to the global burden of disease [1, 2], access to evidence-based SUD treatment can diminish this risk for poor health. Accordingly, improving SUD treatment provision is included in the United Nations’ Sustainable Development Goals for 2030 [3]. However, treatment availability remains severely constrained in South Africa, a country where there is a high prevalence of substance use (SU) and SUDs [4, 5].

Although demographic data on SU are limited in South Africa, nationally 10.3% of the adult population (15 years and older) are estimated to consume alcohol at harmful levels (16.5% of men and 4.6% of women) and 8.6% (13.3% of men and 4.1% of women) are estimated to use illicit drugs [4]. In this context, illicit drug use is mainly driven by cannabis, followed by Mandrax (methaqualone), amphetamine-type stimulants such as methamphetamine, and opiates [4, 5]. Evidence further suggests that a substantial proportion (13.3%) of South Africans meet diagnostic criteria for a SUD [6], with alcohol use disorder being the most common type of SUD experienced [7]. Given these estimates, it is not surprising that South Africa’s burden of disease attributable to alcohol is among the highest in the world, accounting for approximately 3013 disability adjusted life years (DALYS, 95% UI: 2409–3610) per 100 000 people. Although the drug-attributable disease burden is lower than that of alcohol, it is still within the severe range, accounting for an estimated 572 DALYS (UI 471–705) per 100 000 people [8].

Despite the prevalence of SUDs in this setting [5, 6], treatment availability is limited with less than 5% of individuals struggling with SUDs ever accessing treatment [9, 10]. The structural and systemic factors that contribute to this treatment gap are well documented. These include treatment infrastructure constraints, with South Africa’s SUD treatment system limited to 86 treatment sites that provide about 20,000 outpatient and residential treatment episodes annually [11, 12]. The bulk of these services are in the Western Cape province, where the prevalence of SUDs is substantially higher (20.6%) than the national average [6]. Not-for -profit organizations (funded wholly or in part by block grants provided by the Department of Social Development) and free state-managed services account for most of the treatment episodes [11, 13]. While there is a private-for-profit treatment sector, this is only available to the small number of South Africans with private medical insurance or who can afford to pay for these services [13]. Regardless of funding source, SUD treatment is largely non-specific (targeting all types of SUDs) and behavioral in nature [5], with pharmacotherapy only available to individuals who can afford out-of-pocket payments for medication [14]. Together with human resource shortages, these treatment infrastructure constraints contribute to high patient caseloads, long waiting periods for treatment and geographic access barriers [15–17]. Despite recent investments in additional state-funded evidence-based SUD services [18, 19] and workforce development initiatives [17, 20], access to SUD treatment remains limited.

In this context of scarce treatment resources, every opportunity for SUD treatment requires optimisation through providing appropriate and effective care [21–23]. Reports that only 2.3% (± 1%) of South Africans with a past year SUD accessed minimally adequate treatment [24] coupled with patient concerns about the effectiveness of available services [25–27] highlight the need to improve the quality of South Africa’s SUD treatment offering.

System-level data on access to *and* quality of treatment are needed to guide SUD treatment system improvement. While the South African Community Epidemiology Network on Drug Use (SACENDU) has monitored patterns of SUD treatment utilisation for more than 25 years [11, 28], systems-level data on patient experiences of care have rarely been collected [29, 30]. This is a critical gap to address as patients’ perspectives on the SUD treatment experience are likely to influence their engagement in and completion of SUD treatment, which are important predictors of SUD treatment outcomes [31–33]. Further, patients can offer unique insights from their own lived experience into how SUD services can be improved to better meet the needs of other patients [34, 35]. Patients and provider perspectives on necessary components of quality SUD treatment often differ [35], and therefore obtaining the patient perspective on what works in SUD treatment is necessary to guide initiatives aimed at improving the patient’s experience and outcomes of SUD treatment.

Several SUD-specific patient-reported experience measures (PREMs) and patient-reported outcome measures (PROMs) now exist to support the routine collection of these data (see Ref. [31, 34, 36, 37]) including measures developed and validated for the South African treatment context [30, 38]. Examining patient and treatment characteristics associated with poorer performance on these PREMs and PROMs can highlight focal points for interventions such as patient sub-groups reporting more negative experiences of care [32, 36]. However, only a handful of studies, largely from high-income countries, have used PREMS and PROMS for this purpose [32, 36, 39, 40]. Patient characteristics that may influence performance on these measures include age, gender, marital status, race/ethnicity, socio-economic status (employment and income), education, type of substance used, presence of co-occurring mental or physical health problems, treatment expectations, and prior experience of treatment [36, 39, 40]. Treatment characteristics associated with performance on these measures may include intensity of treatment (residential versus outpatient), type of treatment programme, and treatment duration [32].

Although, the pattern of patient and treatment factors associated with PREMs and PROMs is likely to vary between high-income and low-and middle-income countries (LMICs) due to differences in patient population and treatment system profiles, no studies have used patient-reported measures to identify targets for SUD treatment system strengthening in low-and middle-income countries. To address this gap and establish locally relevant targets for SUD treatment improvement initiatives, we explored factors associated with sub-optimal performance on PROMs and PREMs for a treatment population in South Africa, an upper-middle income country. More specifically, this paper aims to identify patient socio-demographic and substance use characteristics as well as treatment factors associated with sub-optimal performance on patient-reported (1) access to, (2) quality and (3) SUD outcomes for the Western Cape province of South Africa’s SUD treatment system.

## Methods

### Study overview and setting

For this paper, we examined patient-reported experience and outcome data collected during 2019 as part of the routine implementation of the Service Quality Measures (SQM) performance measurement system in the Western Cape Province of the country [41]. This system collects patient-level data from 32 treatment sites representing 90% of available treatment sites in the region [41].

The bulk of these treatment sites (n = 28) are dispersed across the eight health subdistricts of the Cape Town metropole, the most densely populated region of the province. The remainder of the treatment sites are in the rural Winelands region of the province. Treatment sites vary in size of treatment population, type of service offering (residential versus outpatient), and funding source [11, 12]. The majority (n = 26) provide outpatient services, with only six providing residential care. Three of the 32 sites (all residential services) are private-for-profit facilities. The remainder of the services are either fully or partially funded through the state. None of these services focus on particular substances of concern (for example, opiates). As part of their funding requirements, all state-funded outpatient facilities offer either motivational enhancement therapy (MET), cognitive behavioral treatment (CBT) or the Matrix model of treatment as evidence-based treatments [13, 18]. Residential programs tend to incorporate elements of MET, CBT and 12-step facilitation.

### SQM implementation and data collection procedures

The SQM system was co-developed with service users and treatment providers in 2013 in response to an identified need [42, 43], before being initially implemented in six pilot sites in 2014 [44]. This co-design process identified strategies for overcoming potential implementation barriers and enhanced provider readiness to adopt the system [43–45]. Over time, system implementation protocols have evolved to enhance feasibility of implementation and its utility to providers [41]. System reach has expanded to 32 sites in the Western Cape as providers grew aware of the benefits of the system for treatment improvement [41, 43].

All SUD treatment sites voluntarily participating in SACENDU are invited to implement the SQM system. Project staff approached treatment managers at these facilities, described the SQM initiative and invited them to participate in system implementation. Facilities had to agree to (1) release staff for initial and ongoing training in the implementation protocols, (2) adopt the performance measurement data collection protocols, and (3) implement the SQM system for at least the minimal data collection period (spanning 1 August through 31 October each year). At the implementing sites, providers complete a standardised two-page SACENDU treatment admission form [22]. On admission, patients are informed about the system and are asked to consent to completing a patient survey about their treatment experiences. Less than 3% of patients decline to participate in the system [41]. At least three weeks after entering treatment, patients are asked to complete the South African Addiction Treatment Services Assessment (SAATSA) [38] that includes several PREMS and PROMs. Patients who leave treatment prematurely often do not complete a SAATSA. This is a barrier for outpatient services, where treatment disengagement is high within the first two weeks of treatment [33]. Protocols require providers to re-contact patients and complete the SAATSA telephonically, but providers often experience difficulties in tracing patients who disengage from services [41]. Finally, providers complete a standardised discharge form on every patient within 30 days of treatment ending [30]. This form gathers information on type of treatment, duration, and clinicians’ perceptions of the patient’s treatment response [33]. Providers can complete this form with the patient or retrospectively, using data extracted from their case files and treatment records. A unique patient identifier allows for data extracted from these three forms to be combined to monitor access, effectiveness, quality, and efficiency of services.

Participating facilities are not financially incentivized to implement the system but are given individualized reports on their performance with data-led recommendations on how they can strengthen their programmes [41]. Providers are more likely to implement the SQM system with fidelity where their leaders and managers mandate implementation and are committed to using system findings for quality improvement [41].

### Measures

The measures used in this paper include patient demographic and substance use variables extracted from the SACENDU admission form, PREMs and PROMs from the SAATSA, and treatment-related variables extracted from the discharge form.

#### Sociodemographic characteristics

We extracted data from the SACENDU form on patients’ age, gender (male versus female), race (Black/African, Coloured (persons of mixed race ancestry), White, or Indian descent), education (completed high school versus not completed high school), and employment status (yes/no).

#### Clinical characteristics

We extracted data from the SACENDU form on primary substance of use (stimulants like methamphetamine and cocaine, opiates, cannabis, methaqualone (Mandrax), and alcohol); presence of polysubstance use (yes/no); frequency of use (daily, 2–6 times per week, weekly, monthly); a continuous measure of number of years of substance use as an indicator of chronicity; and prior experience of SUD treatment (yes/no). We also extracted information from the SACENDU form on the presence of any known (1) physical noncommunicable diseases (NCDs) namely hypertension, cardiovascular disease, diabetes, respiratory disease, liver disease and gastrointestinal disease and separately (2) mental health diagnoses at the time of enrolment into SUD treatment. The SACENDU system does not record specific mental health diagnoses. While specific NCDs are recorded, many of the NCDs have small numbers of cases. We therefore created a dichotomous variable reflecting the presence or absence of any type of comorbid physical noncommunicable disease.

#### Treatment factors

Intensity of treatment provided (residential or outpatient), source of funding for treatment (state versus other) and completion of treatment (yes/no) were extracted from the SACENDU admission and SQM discharge form. Tratment completion was defined as completion of the specified treatment programme.

#### PREMs and PROMs

The SAATSA consists of four PROMs measuring changes in substance use, quality of life, social connectedness, and sexual risk behaviour and two PREMs assessing accessibility and overall quality of services [32]. For this paper, we report on system performance for the access, overall quality, and substance use measures.

The PREM relating to access comprises five items (such as “*The amount of time I had to wait to get services was acceptable to me*” and “*It is easy for me to obtain the treatments offered by this centre*”) with patient responses ranging on a four-point scale from strongly disagree (0) to strongly agree (3). Composite scores, calculated by summing the responses, range between 0 and 15, with higher scores indicating fewer perceived difficulties in accessing services (Cronbach’s alpha = 0.79).

The PREM relating to quality comprises six SAATSA items related to theoretical understandings of person-centred care [32]. Examples of items include “*I have a say in deciding about substance abuse treatment I am receiving here*” and “*The staff at this treatment centre are sensitive to my background.*” Items are scored using the patient response scale described earlier with composite scores ranging between 0 and 18 and higher total scores indicating more positive perceptions of the quality and person-centredness of treatment. A Cronbach’s alpha coefficient of 0.83 was obtained for this measure.

The substance use PROM contains four SAATSA items that are scored using the identical patient response scale. Examples of items include “*As a result of the treatment I have received I am less likely to use alcohol or other drugs*” and “*The treatment centre is helping me to recover from using alcohol and drugs*.” Composite scores range between 0 and 12, with higher scores indicating more positive outcomes. For this scale, the Cronbach’s alpha coefficient was 0.81.

### Analyses

All analyses were conducted using Stata 16 (Stata Corporation, College Station, TX, USA), with significance set at *p* < 0.05. We used Stata’s survey analysis platform to account for clustering by treatment site. In 2019, the SQM system collected 3318 SACENDU admission, 1720 SAATSA, and 2569 SQM discharge forms. Of these, we were able to link a SACENDU admission, SAATSA, and SQM discharge form for 1139 cases. Of these, 42 cases had too much missing data from the SAATSA items to allow for the calculation of the PROM or PREMs. As the data seemed to be missing at random, and because these cases represented ~ 3% of the sample with linked data, we decided to removing these cases using listwise deletion. The final dataset therefore included 1097 observations. We used adjusted Wald tests and t-tests to compare the demographic characteristics of the realized sample against that of the SACENDU treatment population for the specified reporting period. Next, as scores on each of the SAATSA scales were positively skewed, we recoded these into optimal versus sub-optimal performance after transforming the composite scale scores into percentage scores indicating the average percentage of the score relative to the total possible score. For the access and quality PREMs and substance use PROM, scores < 80% were coded as “*substantial difficulties in accessing SUD treatment*”, “*suboptimal quality of treatment*”, or “*suboptimal SUD outcomes,*” respectively. Next, we conducted logistic regression (for categorial variables) and linear regression (for continuous variables) analyses to identify and describe possible relationships between socio-demographic, substance use, and treatment factors and sub-optimal performance on three patient-reported measures (access, quality, substance use outcomes). Variables associated with these measures at *p* < 0.10 were entered in multivariable logistic regression models that adjusted for gender, age and race. Results are reported as adjusted odds ratios (aOR) with 95% confidence intervals (CIs).

## Results

### Sample characteristics

The 1097 cases included in the analyses represent 33.1% of the total SUD treatment population at participating sites. All 32 treatment sites are represented in the final sample. Table 1 compares the demographic characteristics of the included cases with the characteristics of cases not included in the analysis and the total treatment population. In the final sample, 26.3% of the cases were female, 7.7% were Black African and 80.4% were Coloured (a designated race in South Africa comprising people of mixed-race ancestry), the mean age was 33.4 years (SD = 10.1), 51.4% had completed high school, and 73.7% were unemployed. Patients who were completed the SAATSA and were included in the analyses were more likely to have completed high school than those did not (51.4% vs 46.3%, *p* = 0.032). In this sample and the treatment population, the two most frequently reported substances used were stimulants (40.9% of the sample versus 39.1% of the treatment population), followed by opiates (29.6% of the sample versus 25.3% of the treatment population). Patients reporting cannabis as their primary substance were slightly under-represented in the analytic sample compared to the overall treatment population (6.1% vs. 12.9%). In terms of treatment intensity, patients who completed the SAATSA and were included in the analyses were more likely to have obtained residential treatment than those excluded from the analyses (37.3% vs 24.9%, *p* = 0.002; Table 1).

**Table 1:** Baseline sociodemographic, substance use and treatment characteristics of cases included in the sample, compared to the overall treatment population

Variable	Included in the sample (N = 1097) n (%)	Not included in the sample (N = 2221) n (%)	Overall treatment population (N = 3318) n (%)
Gender			
Male	808 (73.7)	1572 (70.8)	2380 (71.7)
Female	289 (26.3)	649 (29.2)	938 (28.3)
Race			
Black African	84 (7.7)	238 (10.7)	322 (9.7)
Coloured^a^	882 (80.4)	1755 (79.0)	2637 (79.5)
White	103 (9.1)	218 (9.8)	321 (9.7)
Indian	31 (2.8)	11 (0.5)	42 (1.3)
Age (M, SD) in years	33.4 (10.1)	32.7 (9.8)	33.1 (9.9)
Education			
Completed school	564 (51.4)	1028 (46.3)*	1592 (48.0)
Not completed school	533 (48.6)	1193 (53.7)	1726 (52.0)
Employment			
Employed	289 (26.3)	624 (28.1)	913 (27.5)
Unemployed	808 (73.7)	1597 (71.9)	2405 (72.5)
Primary substance			
Stimulants	449 (40.9)	848 (38.2)	1297 (39.1)
Opiates	325 (29.6)	515 (23.2)	840 (25.3)
Cannabis	67 (6.1)	362 (16.3)*	429 (12.9)
Methaqualone	93 (8.5)	213 (9.6)	306 (9.2)
Alcohol	163 (14.9)	282 (12.7)	445 (13.4)
Treatment intensity			
Residential	406 (37.3)	553 (24.9)**	959 (28.9)
Outpatient	688 (62.7)	1668 (75.1)	2356 (71.0)

^a^“ Coloured” refers to people of mixed race ancestry- a designated population group in South Africa

^*^significant at p < 0.05 ** significant at p < 0.01

### Patient-reported access to, quality of, and outcomes of treatment

Of the patients who completed the SAATSA measure, 37.9% (95% CI 29.7%; 46.8%) erceived substantial difficulties in accessing SUD treatment, 28.8% (95% CI: 23.7%; 34.4%) perceived SUD treatment to be of suboptimal quality, and 31.1% (95% CI 24.4%; 38.8%) reported suboptimal SUD outcomes.

### Variables associated with greater perceived difficulty in accessing SUD treatment

Factors associated with perceived access to SUD treatment in unadjusted analyses included race, presence of co-occurring physical noncommunicable diseases or mental health problems, type of substance used, polysubstance use, chronicity of substance use, the source of payment for treatment and the intensity of treatment (Table 2). In multivariate analyses, several of these factors remained independently associated with perceived access to treatment. Patients who self-identified as Coloured or White were significantly less likely to perceive difficulties in accessing SUD treatment than participants who self-identified as Black African (aOR = 0.23; 95% CI 0.11–0.51; aOR = 0.28; 95% CI 0.11–0.71, respectively). Patients with comorbid mental health problems had significantly greater odds of experiencing suboptimal access to treatment than patients without these difficulties (aOR = 1.91; 95% CI 1.16–3.14). The odds of experiencing suboptimal access to SUD treatment was elevated for patients in residential treatment (aOR = 1.69; 95% CI 1.08–2,64) and with chronicity of substance use (aOR = 1.02; 95% CI 1.02–1.05; Table 2).

**Table 2:** Sociodemographic, substance use and treatment variables associated with greater perceived difficulty in accessing SUD treatment (n = 1097)

Variable	Poor performance on SAATSA Access Scale *n (%)*	Unadjusted association		Adjusted association	
		OR^a^	95% CI^b^	aOR^c^	95% CI
Gender					
Male	318 (39.1)	Reference		Reference	
Female	99 (35.0)	0.84	0.59–1.19	0.89	0.69–1.27
Race					
Black African	44 (57.1)	Reference		Reference	
Coloured^d^	308 (34.9)	0.22	0.12–0.40***	0.23	0.11–0.51***
White	60 (41.6)	0.34	0.21–0.57***	0.28	0.11–0.71**
Indian	5 (47.6)	1.24	0.10–14.96	1.24	0.11–12.70
Age (M, SE) in years	33.7 (0.58)	1.01	0.99–1.03	0.98	0.96–1.00
Education					
Not completed school	174 (33.0)	0.66	0.41–1.07	–	
Employment					
Employed	127 (44.2)	1.42	0.99–2.05	1.13	0.71–1.75
Comorbid physical noncommunicable disease	56 (50.0)	1.84	1.26–2.70***	1.84	0.99–3.52
Comorbid mental health	51 (51.0)	1.92	1.01–3.88*	1.91	1.16–3.14**
Primary substance					
Stimulants	145 (35.1)	0.58	0.40–0.84**	0.84	0.46–1.58
Heroin	96 (36.5)	0.51	0.35–0.74**	1.03	0.45–2.36
Cannabis	23 (32.1)	0.61	0.38–0.98*	0.81	0.40–1.62
Methaqualone	34 (40.0)	0.71	0.35–1.44	1.04	0.43–2.23
Alcohol	73 (48.3)	Reference		Reference	
Polysubstance use	227 (35.0)	1.32	1.01–1.75*	1.26	0.89–1.79
Chronicity: years of use (*M, SE*)	15.12 (0.79)	1.02	1.00–1.03*	1.02	1.02–1.05**
Frequency of use					
Daily	276 (35.7)	2.41	0.52–1.10	–	
2–6 times per week	84 (42.2)	3.17	0.69–14.57		
Weekly	10 (37.0)	2.54	0.53–12.28		
Monthly	3 (18.8)	Reference			
Prior SUD treatment No	190 (37.7)	Reference			
Yes	227 (38.2)	1.02	0.79–1.32	–	
Treatment intensity					
Outpatient	215 (31.6)	Reference		Reference	
Residential	202 (48.4)	2.03	1.40–2.96**	1.69	1.08–2.64**
State-funded treatment					
Yes	286 (34.3)	0.53	0.29–0.96*	0.90	0.53–1.54
No	131 (49.8)	Reference		Reference	

^a^*OR* odds ratio, ^b^*95% CI* 95% confidence interval; ^c^*AOR* adjusted odds ratio; ^d^“ Coloured” refers to people of mixed race ancestry- a designated population group in South Africa

* significant at p < 0.05, ** significant at p < 0.01, ***significant at p < 0.001

### Variables associated with patient perceptions of suboptimal SUD treatment quality

In unadjusted analyses, race, polysubstance use, chronicity of substance use, and prior experience of SUD treatment were associated with perceptions of suboptimal quality of SUD treatment (Table 3). In multivariate analyses, race remained independently associated with suboptimal SUD treatment quality. Patients who self-identified as Coloured were significantly less likely to perceive SUD treatment as being of poorer quality than those who were Black African (aOR = 0.32; 95% CI 0.16–0.66). The odds of perceiving SUD treatment to be of suboptimal quality increased as years of substance use increased (aOR = 1.03; 95% CI 1.00–1.05). Patients with comorbid mental health problems (aOR = 2.08; 95% CI 1.24–3.49), and who had previous experience of SUD treatment (aOR = 1.79; 95% CI 1.42–2.51) were more likely to report experiencing treatment of suboptimal quality (Table 3).

**Table 3:** Sociodemographic, substance use and treatment variables associated with patient perceptions that SUD treatment was of suboptimal quality (n = 1097)

Variable	Poor performance on SAATSA Quality Scale *n (%)*	Unadjusted association		Adjusted association	
		OR^a^	95% CI^b^	aOR^c^	95% CI
Gender					
Male	241 (29.6)	Reference		Reference	
Female	77 (27.1)	0.89	0.65–1.21	0.76	0.51–1.14
Race					
Black African	35 (44.9)	Reference		Reference	
Coloured^d^	226 (25.5)	0.42	0.30–0.60***	0.32	0.16–0.66**
White	57 (41.5)	0.87	0.59–1.28	0.57	0.30–1.07
Indian	6 (50.0)	1.23	0.34–4.43	3.62	0.39–32.25
Age (M, SE) in years	34.11 (0.59)	1.01	0.99–1.03	0.97	0.96–1.02
Education					
Not completed school	135 (25.3)	0.71	0.46–1.09	–	
Employment					
Employed	100 (35.0)	0.68	0.43–1.09	–	
Comorbid physical noncommunicable disease	36 (33.0)	1.63	0.92–1.83	1.14	0.78–1.76
Comorbid mental health	46 (45.1)	2.31	0.97–5.52	2.08	1.24–3.49**
Primary substance					
Stimulants	112 (26.9)	0.63	0.38–1.64	–	
Heroin	80 (26.4)	0.61	0.34–1.09		
Cannabis	19 (30.7)	0.76	0.43–1.32		
Methaqualone	20 (23.5)	0.53	0.16–1.73		
Alcohol	55 (36.9)	Reference			
Polysubstance use	173 (26.7)	1.33	1.01–1.77*	1.25	0.88–1.77
Chronicity: years of use (*M, SE*)	15.39 (0.72)	1.02	1.01–1.04**	1.03	1.00–1.05*
Frequency of use					
Daily	199 (25.8)	1.22	0.32–4.08	–	
2–6 times per week	77 (38.1)	2.16	0.52–9.02		
Weekly	6 (23.1)	1.05	0.17–6.35		
Monthly	4 (22.2)	Reference			
Prior SUD treatment					
No	154 (26.0)	Reference		Reference	
Yes	164 (32.4)	1.36	1.13–1.64**	1.79	1.28–2.51***
Treatment intensity Outpatient	176 (25.8)	Reference		–	
Residential	142 (34.1)	1.49	0.77–2.90		
State-funded treatment					
Yes	217 (26.0)	0.57	0.25–1.33	–	
No	101 (38.1)	Reference			

^a^*OR* odds ratio, ^b^*95% CI* 95% confidence interval, ^c^*AOR* adjusted odds ratio, ^d^“ Coloured” refers to people of mixed race ancestry- a designated population group in South Africa

*significant at p < 0.05, **significant at p < 0.01, ***significant at p < 0.001

To better understand the association between race and perceptions of SUD treatment quality, we explored associations between race and the individual items comprising this PREM. Notably, a higher proportion of Black African patients reported provider insensitivity to cultural background (16.8%) compared to Coloured (5.9%), White (6.2%) and Indian (5.6%) patients (*p* = 0.002). Similarly, when we explored the SAATSA item assessing patient perceptions of being treated with respect, significantly more Black African patients reported not being treated with respect (10.8%) compared to Coloured (4.8%), White (5.1%) and Indian (3.2%) patients (*p* = 0.041). Black African patients were also more likely to report a lack of involvement in their SUD treatment planning (12.6%) compared to Coloured (4.7%), White (4.2%) and Indian (3.6%) patients (*p* = 0.035).

### Factors associated with patient perceptions of suboptimal SUD outcomes

In unadjusted analyses, age, polysubstance use, comorbid mental health problems, failure to complete treatment, and perceptions of suboptimal treatment quality were the only variables significantly associated with patient perceptions of suboptimal SUD outcomes (Table 4). In multivariate analyses, women were significantly less likely to report suboptimal SUD outcomes than men (aOR = 0.57; 95% CI 0.35–0.93). The odds of experiencing suboptimal treatment outcomes were greater amongst patients with comorbid mental health problems (aOR = 1.41; 95% CI 1.02–2.05) and for patients reporting polysubstance use (aOR = 1.68; 95% CI 1.11–2.55). Patients who did not complete their full treatment programme had triple the odds of reporting poorer outcomes than those who completed their programme as planned (aOR = 3.03; 95% CI 1.33–6.86). Patients who perceived SUD treatment to be of suboptimal quality had substantially elevated odds of reporting suboptimal treatment outcomes (aOR = 20.82; 95% CI 13.59–31.88; Table 4).

**Table 4:** Sociodemographic, substance use and treatment variables associated with patient perceptions of suboptimal SUD treatment outcomes (n = 1097)

Variable	Poor performance on substance use PROM *n* (%)	Unadjusted association		Adjusted association	
		OR^a^	95% CI^b^	aOR^c^	95% CI
Gender					
Male	265 (32.3)	Reference		Reference	
Female	82 (28.8)	0.84	0.61–1.19	0.57	0.35–0.93*
Race					
Black African	30 (37.9)	Reference		Reference	
Coloured^d^	265 (29.8)	0.69	0.42- 1.15	1.24	0.38–4.08
White	51 (40.2)	1.10	0.75–1.61	2.07	0.76–5.63
Indian	1 (8.3)	0.15	0.02–1.19	0.11	0.00–5.53
Age (M, SE) in years	33.31 (0.59)	1.01	1.00–1.02*	1.01	0.99–1.04
Education					
Not completed school	157 (29.3)	0.83	0.62–1.11	–	
Employment					
Employed	93 (32.3)	1.06	0.57–1.95	–	
Comorbid physical noncommunicable disease	40 (38.5)	1.51	0.66–3.42	–	
Comorbid mental health	40 (35.7)	1.32	1.06–1.66**	1.41	1.02–2.05*
Primary substance					
Stimulants	121 (28.8)	0.94	0.46–1.92	-	
Heroin	99 (32.5)	1.12	0.52–2.39		
Cannabis	23 (37.1)	1.37	0.82–2.28		
Methaqualone	21 (24.7)	0.76	0.17–3.52		
Alcohol	46 (30.1)	Reference		Reference	
Polysubstance use	185 (28.4)	1.56	1.19–2.05***	1.68	1.11–2.55*
Chronicity: years of use (*M, SE*)	14.66 (0.59)	1.01	0.99–1.02	0.98	0.96–1.01
Frequency of use					
Daily	199 (25.8)	0.89	0.31–2.50	–	
2–6 times per week	77 (38.1)	1.22	0.34–4.46		
Weekly	6 (23.1)	0.54	0.09–3.22		
Monthly	4 (22.2)	Reference			
Prior SUD treatment					
No	154 (26.0)	Reference		Reference	
Yes	164 (32.4)	1.18	0.91–1.54	1.08	0.73–1.61
Treatment intensity Outpatient	229 (33.3)	Reference		–	
Residential	118 (28.2)	0.78	0.38–1.61		
State-funded treatment					
Yes	262 (31.3)	0.98	0.43–2.21	–	
No	85 (31.7)	Reference			
Did not complete treatment	21 (46.7)	1.97	1.06–3.68*	3.03	1.33–6.86**
Treatment quality perceived as suboptimal	230 (73.0)	16.31	0.97–24.25***	20.82	13.59–31.88***

^a^*OR* odds ratio, ^b^*95% CI* 95% confidence interval, ^c^*AOR* adjusted odds ratio, ^d^“ Coloured” refers to people of mixed race ancestry- a designated population group in South Africa

*significant at p < 0.05, **significant at p < 0.01, ***significant at p < 0.001

## Discussion

To the best of our knowledge, no other studies from LMICs have used data from patient-reported measures to identify targets for SUD treatment system strengthening. To address this gap, we describe the performance of the Western Cape’s SUD treatment system on patient-reported measures of treatment access, quality, and outcomes. With more than a third of participants reporting substantial difficulties in accessing SUD treatment and more than a quarter describing suboptimal quality of care, our findings confirm that access to quality SUD treatment remains challenging for a substantial proportion of people with SUDs despite system-level efforts to scale up the availability of evidence-based SUD treatment in this province [18–20]. Findings that almost a third of patients report unsatisfactory SUD outcomes, with patient perceptions of suboptimal treatment quality being the variable most strongly associated with these poor outcomes, suggest that the Western Cape is unlikely to see a return on its investment in SUD treatment without systems-level interventions to improve the quality of existing SUD treatment services.

Our findings not only underscore the considerable need for SUD treatment quality improvement in this region, but also highlight disparities in treatment experiences and outcomes that require urgent intervention to ensure equitable access to quality treatment. Notably, patients who self-identified as Black African were more likely to report inadequate access to and suboptimal quality of SUD treatment than patients from other racial groups, reflecting persisting racial disparities in the country’s SUD services. Interventions that improve access to and the experience of SUD treatment for people who identify as Black/African require prioritisation to ensure health equity and redress apartheid’s legacy of substandard health and social services for Black/African citizens. With less than one percent of SUD facilities located in predominantly Black/African communities and evidence that the cost and time burden associated with obtaining SUD treatment is higher for patients from Black/African communities than from communities well-served by treatment facilities [46], it is not surprising that patients of Black/African descent report more difficulties in obtaining treatment than other groups. Redressing these inequities will require treatment planners to locate new SUD services in underserved Black/African communities and consider the geospatial redistribution of existing SUD services in relation to unmet treatment need.

Our findings further suggest insufficient provision of culturally tailored, patient-centred SUD services. Specifically, patients of Black/African descent were more likely to report provider insensitivity to their (cultural) background not being treated with respect and lack of involvement in treatment planning. These findings are not surprising given that only three of the 32 sites were able to provide SUD services in isiXhosa, the most frequently spoken African language in the Western Cape. Associations between absence of culturally tailored, person-centred SUD treatment and suboptimal SUD outcomes among African migrant populations and racial minorities in high-income country studies [47, 48] underscore the need for systems-level interventions to improve Black/African patients’ experience of care. Interventions that increase the cultural and language diversity of SUD providers in the Western Cape, expand SUD workforce training efforts to include cultural explanations of SUDs, and implement training to not only improve providers’ cultural competence but also address their unconscious biases towards people of Black/African descent may help improve the SUD treatment experience of Black/African patients. The latter may be especially relevant as institutional racism continues to cloud the interactions of patients and providers from different race groups in South Africa [49].

Disparities in access to and outcomes of SUD treatment for patients with comorbid mental disorders also emerged as a target for quality improvement. Findings indicate substantially greater risk of suboptimal access to, quality, and outcomes of SUD treatment among patients with comorbid mental and substance use disorders (MSUD) than patients with SUD alone. This study provides the first empirical evidence to support long-standing concerns about the relevance and appropriateness of South Africa’s SUD services for people with comorbid MSUD [50, 51]. South Africa’s publicly funded SUD treatment system rarely provides integrated SUD and mental health treatment, largely due to a lack of intersectoral collaboration between the separate SUD and mental health treatment systems that has resulted in SUD providers often lacking the training and skills to address comorbid mental disorders among people with SUDs [51, 52]. While SUD providers do refer patients with comorbid conditions to external mental health services, patients struggle to access chronically under-resourced public sector services due to long waiting periods, provider stigma towards people with SUDs, and other patient barriers [51, 53].

With at least 50% of patients estimated to have comorbid MSUD [54, 55], publicly funded SUD services must introduce integrated MSUD services to optimise the overall impact of SUD treatment. Evidence-based integrated MSUD treatments exist, with some already developed for the South African context [56, 57]. As social work professionals and lay addiction counsellors with limited training in mental disorders comprise the bulk of South Africa’s SUD providers [51], implementing these programmes will require additional workforce development. With specialist mental health providers being a scarce resource in the country [53], expanding SUD treatment teams to include specialist providers is not realistic. Instead, we recommend drawing on the experiences of the global mental health movement which has demonstrated that structured evidence-based treatments for common mental disorders (like depression, anxiety and psychological trauma) can be task-shifted to non-specialist providers with adequate training and support [58]. Local studies have shown that these task-shifted interventions are feasible to implement and acceptable to providers and patients with comorbid MSUD [59, 60]. Should SUD treatment planners implement this recommendation, current SUD workforce development initiatives [20] could be expanded to include capacitation of SUD providers on the necessary core competencies.

Our findings further suggest that risk of experiencing suboptimal access to and quality of SUD treatment is greater amongst patients with lengthier histories of substance-related problems than those with less complex clinical presentations. This may be due to the configuration of the Western Cape’s SUD treatment system. Here, SUD treatment is provided mainly through outpatient services, with a limited number of residential facilities available. In this study, patients reported greater difficulty in accessing residential than outpatient services. The restricted supply of residential SUD treatment, the level of care most appropriate for individuals with chronic and severe SUDs [33], may explain why individuals with chronic problems experience greater difficulties in accessing care. Further, most SUD programmes in this region are of limited duration (21–28 days). The responsiveness of such programmes to the multiple needs of individuals with chronic SUDs is questionable. Although the intention is for patients to access continued support post-treatment, through structured aftercare or mutual/self-help support programmes, little is known about existing continued care services. A better understanding of the structure and content of these continued support programmes (as well as patient perceptions of their utility) is needed to guide efforts to improve the appropriateness of SUD services for people with chronic SUDs.

There are some limitations to consider when interpreting these findings. With SQM system implementation being restricted to the Western Cape, findings may not be generalizable to other provinces. The type of SUD treatment provided is similar across the country, but there are interprovincial differences in the prevalence of SUD and the sociodemographic and substance use characteristics of people who use substances [11] that are likely to influence experiences and responses to treatment. Furthermore, our analyses are limited to a third of the total number of treatment episodes at participating sites. Although baseline characteristics of the included cases and overall treatment population seemed similar, residential treatment episodes were overrepresented in our sample (likely due to high rates of attrition from outpatient treatment), which may have influenced findings. In addition, as analyses were limited to variables included in the SQM system, we were unable to assess other patient (e.g. motivation or treatment expectations) or other treatment-related factors (such as type of treatment programme or counsellor characteristics) known to be associated with patient perceptions and outcomes of SUD interventions in this setting [61]. In response to these system limitations, we are now collecting detailed information from each participating SUD treatment facility on the type of treatment provided and composition of SUD providers that will enable a more nuanced and comprehensive analysis of the correlates of patient-reported experiences and outcomes of SUD treatment in this setting.

## Conclusion

This study is among the first to use routinely collected patient-reported measures to identify targets for SUD treatment system improvement in a LMIC. With a substantial proportion of participants reporting difficulties in accessing SUD treatment, suboptimal quality of care, and unsatisfactory SUD outcomes, there is a clear need for SUD treatment quality improvement initiatives in this setting. Our findings also highlight the need for targeted efforts to ensure equitable access to quality treatment for patients of Black/African descent and those with more complex and severe clinical presentations. We propose a variety of systems-level interventions to improve the quality of care, and ultimately the SUD treatment outcomes, for these patient subgroups that include reconsidering the spatial distribution of SUD services to improve access to SUD treatment in Black/African communities, workforce development interventions to build cultural competency and address unconscious racial bias among providers, building provider competency to deliver evidence-based integrated MSUD services (and supporting them in programme implementation), and re-looking at the role of continued care and support services for patients with more complex and chronic SUDs. Although these findings are specific to the South African treatment context, our findings demonstrate the benefits of collecting PREMs and PROMs through a routinely implemented performance measurement system and how these data can be used to guide SUD treatment improvement initiatives in resource-limited contexts. The PREMs and PROMs contained in the SQM system could easily be adapted for implementation in other low-and middle-income countries and are likely to be highly acceptable to patients and treatment facilities, provided that the process of adapting the measures for cultural fit follows a co-design process.

## Data Availability

Data and materials are available on request from the corresponding author.

## Abbreviations

LMIC: Low-and middle-income country
PREM: Patient-reported experience measure
PROM: Patient-reported outcome measure
SAATSA: South African Addiction Treatment Services Assessment
SACENDU: South African Community Epidemiology Network on Drug Use
SQM: Service quality measures
SUD: Substance use disorder
